# Music to move persons with Parkinson’s disease: a personalized approach

**DOI:** 10.1007/s00415-021-10615-5

**Published:** 2021-05-22

**Authors:** Susanne Ten Holter, Jorik Nonnekes, Bastiaan Bloem

**Affiliations:** 1 Department of Neurology, Donders Institute for Brain, Cognition and Behaviour, Radboud University Medical Centre, PO Box 9101, 6500 HB Nijmegen, The Netherlands; 2 Department of Rehabilitation, Donders Institute for Brain, Cognition and Behaviour, Radboud University Medical Centre, Nijmegen, The Netherlands

## Introduction

Gait impairments, and freezing of gait (FOG) in particular, are common and disabling for many people with Parkinson’s disease (PD) [1]. During FOG episodes, patients feel as if their feet are suddenly being ‘glued to the floor’ [2]. Treatment can be challenging but remains critically important as a good mobility is vital for independence and quality of life. Optimal management involves both pharmacological and non-pharmacological interventions. Non-pharmacological interventions include the use of compensation strategies, which should be tailored to each individual’s preferences and abilities [3, 4]. Clinical experience suggests that what works for one patient may not work for the next, but there is still relatively little documentation of this individualized response to non-pharmacological interventions. Here, we illustrate the importance of personalizing non-pharmacological interventions, and also demonstrate the value of objectively evaluating the effects of dopaminergic medication.

## Case

A 79-year-old man with a 12-year history of PD was seen at our outpatient clinic because of disabling FOG that occurred several times a day, particularly when walking in small spaces, when starting to walk, or during turning. He received levodopa/benserazide 325.5 mg four times daily, ropinirole 2 mg twice daily, rivastigmine 9.5 mg once daily, and clozapine 6.25 mg twice time daily because of mild hallucinations, in line with an advanced stage of PD. He experienced no subjective benefit of dopaminergic medication on overall symptoms, and particularly not on FOG. He noticed, however, an improved ability to move when listening to specific music tracks. In his experience, the greatest subjective improvements would occur when listening to ‘*Che farò senza Euridice*’ of Gluck’s opera *Orfeo ed Euridice*. We decided to take this to the test. During examination in a regular ON state, normal walking was quite good, but making a rapid turn caused marked FOG (video 1). When playing the patient’s favorite music, he was able to rise from a chair without arm support, his steps became larger and faster, his posture straightened and turning became easier, even allowing him to dance without external support (video 2). During some of the turning movements, he still manifested brief FOG episodes, but not as long-lasting as when moving without music. Other music pieces also afforded improvements in walking, but the effect was less pronounced. Remarkably, although the patient denied experiencing any benefits from dopaminergic medication, his symptoms markedly worsened as the medication effects gradually wore off. During this OFF state, he was no longer to rise from a chair without help (video 3). Pronounced FOG episodes were seen, sometimes lasting several minutes. Walking was only possible using a wheeled walker, and was improved when combining this walker with his favorite music. Following the in-house film recordings described here, we gradually increased the levodopa/benserazide dose to 375 mg five times a day, which remarkable improved daily life functioning, to an extent where FOG had largely disappeared. Moreover, his overall functioning (both physically and mentally) also improved, and wearing OFF was better recognized and clearly reduced.


Walking in ON state, FOG when turning at the same place (MP4 25904 KB)ON state with music and making dancing moves there is a further improvement with straight posture and flowing turns without the need for a walker (MP4 48512 KB)OFF state: he can’t rise from a chair without help, he has profound FOG. Using a walker alone doesn’t help him to start walking. Playing music, already when concentrating on the music to come, there is an improvement on posture, steps, speed and FOG (MP4 56797 KB)

The importance of the case report is twofold. First, listening to music can serve as a compensation strategy to optimize walking and reduce FOG. Importantly, the subjective effect was greatest when listening to his favorite music, suggesting that an emotional component (presumably by activation of the limbic system) was a contributing factor. Further working mechanisms may involve the music acting as an external rhythmic cue, enabling a shift from automatic to goal-directed gait control [5]. This could also explain why many patients indicate that certain rhythms work better for them than others, because cueing frequencies must be tailored to the patient’s individual needs and abilities [6]. Moreover, compared to regular walking, dancing movements likely involve alternative motor programs that are less dependent on automatized movement generation by the basal ganglia. Second, although our patient experienced no subjective benefits from dopaminergic medication at home, we actually observed a substantial difference between the dopaminergic ON and OFF states during formal testing in the clinic. Recognizing ON and OFF states and appreciating the effect of dopaminergic medication can be difficult for patients, particularly after a long disease duration. Objective verification of the treatment response can then be helpful, if needed by maximizing the contrast testing patients in both a practically defined OFF state and a clear ON state, after intake of a supramaximal levodopa dose [7]. Understanding the actual degree of dopaminergic responsiveness helps to optimize the pharmacotherapy regime, and this was also illustrated here: increasing the levodopa dose improved FOG in daily life. Our patient benefited most from the combined effect of both medication, favorite music and a walker. As basal ganglia circuitries are presumably still involved in the production of dancing movements, optimization of dopaminergic treatment probably has a complementary effect. This leads to our conclusion that optimal FOG management typically requires a multifaceted and personalized approach.
